# The Interaction of Bluetongue Virus VP6 and Genomic RNA Is Essential for Genome Packaging

**DOI:** 10.1128/JVI.02023-18

**Published:** 2019-02-19

**Authors:** Po-Yu Sung, Robert Vaughan, Shah Kamranur Rahman, Guanghui Yi, Adeline Kerviel, C. Cheng Kao, Polly Roy

**Affiliations:** a Faculty of Infectious and Tropical Diseases, London School of Hygiene and Tropical Medicine, London, United Kingdom; b Biotechnology Program, Indiana University Bloomington, Bloomington, Indiana, USA; c Department of Molecular and Cellular Biochemistry, Indiana University Bloomington, Bloomington, Indiana, USA; Loyola University Chicago

**Keywords:** genome packaging, RNA-protein interaction, double-stranded RNA virus

## Abstract

The genomes of the *Reoviridae*, including the animal pathogen bluetongue virus (BTV), are multisegmented double-stranded RNA (dsRNA). During replication, single-stranded (ss) positive-sense RNA segments are packaged into the assembling virus capsid, triggering genomic dsRNA synthesis. However, exactly how this packaging event occurs is not clear. A minor capsid protein, VP6, unique for the orbiviruses, has been proposed to be involved in the RNA-packaging process. In this study, we sought to characterize the RNA binding activity of VP6 and its functional relevance. A novel proteomic approach was utilized to map the ssRNA/dsRNA binding sites of a purified recombinant protein and the genomic dsRNA binding sites of the capsid-associated VP6. The data revealed that each VP6 protein has multiple distinct RNA-binding regions and that only one region is shared between recombinant and capsid-associated VP6. A combination of targeted mutagenesis and reverse genetics identified the RNA-binding region that is essential for virus replication. Using an *in vitro* RNA-binding competition assay, a unique cell-free assembly assay, and an *in vivo* single-cycle replication assay, it was possible to identify a motif within the shared binding region that binds BTV ssRNA preferentially in a manner consistent with specific RNA recruitment during capsid assembly. These data highlight the critical roles that this unique protein plays in orbivirus genome packaging and replication.

**IMPORTANCE** Genome packaging is a critical stage during virus replication. For viruses with segmented genomes, the genome segments need to be correctly packaged into a newly formed capsid. However, the detailed mechanism of this packaging is unclear. Here we focus on VP6, a minor viral protein of bluetongue virus, which is critical for genome packaging. We used multiple approaches, including a robust RNA-protein fingerprinting assay, to map the ssRNA binding sites of recombinant VP6 and the genomic dsRNA binding sites of capsid-associated VP6. By these means, together with virological and biochemical methods, we identify the viral RNA-packaging motif of a segmented dsRNA virus for the first time.

## Abstract (precis)

Genome packaging is a critical stage during virus replication. For viruses with segmented genomes, the genome segments need to be correctly packaged into a newly formed capsid. However, the detailed mechanism of this packaging is unclear. Here we focus on VP6, a minor viral protein of bluetongue virus, which is critical for genome packaging. We used multiple approaches, including a robust RNA-protein fingerprinting assay, to map the ssRNA binding sites of recombinant VP6 and the genomic dsRNA binding sites of capsid-associated VP6. By these means, together with virological and biochemical methods, we identify the viral RNA-packaging motif of a segmented dsRNA virus for the first time.

## INTRODUCTION

Bluetongue virus (BTV) is an important animal pathogen and the prototype of the genus *Orbivirus*, which belongs to the *Reoviridae* family. The BTV particle has two capsids, an outer capsid and an inner capsid, the latter of which is also called the core. The outer capsid contains proteins VP2 and VP5 to facilitate virus entry through the cellular membrane and the release of the core into the cytoplasm. The icosahedral core is principally composed of two proteins, VP7 and VP3, which are arranged in two layers. VP3 encloses the viral genome of 10 double-stranded RNA (dsRNA) segments (S1 to S10). In addition, the core contains three minor proteins: the polymerase (VP1), the capping enzyme (VP4), and VP6, an essential structural protein of 36 kDa with RNA binding and ATP binding activity. VP6 is unique to the *Orbivirus* genus within the *Reoviridae* family.

Upon entry, core particles become transcriptionally active, producing and extruding single-stranded positive-sense RNAs (ssRNAs) through the local channels at the 5-fold axis, without further disassembly. These ssRNAs then act both as mRNAs for viral protein synthesis and as templates for nascent genomic RNA synthesis. Our current understanding is that the 10 newly synthesized ssRNA segments are first combined via specific intersegment RNA-RNA interactions to form RNA complexes of all 10 segments. The RNA complexes of 10 segments are then packaged together with VP1, VP4. and VP6 into the assembling VP3 capsid layer (1–4). Genomic dsRNA molecules are subsequently synthesized within this assembled particle (known as the “subcore”) prior to encapsidation by the VP7 layer, leading to robust core particle formation (5).

VP1 polymerase and capping enzyme VP4 are likely to be located beneath the VP3 layer at or near the 5-fold axis of icosahedral symmetry to facilitate the release of newly synthesized transcripts (6, 7). However, the exact location of VP6 is not yet clear, although VP6 has specific binding affinity for VP3 and this interaction has been shown to be important for viral ssRNA packaging and replication (8). Using reverse genetics (RG), we have shown that VP6 is essential for BTV replication and that modified BTV strains lacking VP6 do not replicate in normal cells but only in a VP6 helper cell line (9). Further, when VP6-deficient viruses were grown in VP6 helper cells and used for infection of normal cells, viral proteins were synthesized and assembled as empty particles without the viral genome. These data suggest that VP6 may be responsible for genome packaging (10, 11).

The smallest core-associated protein, VP6 (328 amino acids [aa]), has high binding affinity for both ssRNA and dsRNA species, suggesting that it is closely associated with the viral genome (12, 13). VP6 was previously suggested to be an RNA helicase, despite poor homology with known helicases (14). The current hypothesis is that VP6 assists in ssRNA packaging into the viral core through the interaction with VP3 (1, 8, 15). However, questions concerning the definition of the sites that bind viral ssRNAs, whether this is specific for BTV RNAs and, if so, how VP6 interacts with genomic dsRNA remain to be addressed.

In this study, we used RNA cross-linking and peptide fingerprinting (RCAP) to identify the RNA binding sites of VP6 using both a recombinant VP6 protein (reVP6) and VP6 in purified viral cores. The data demonstrate that multiple regions of reVP6 and core-associated VP6 interact with both ssRNA and dsRNA but that each source of VP6 had a largely unique RNA binding profile, with only one region in common. Mutagenesis of residues within the mapped RNA-binding regions followed by virus recovery using the RG system demonstrated that the VP6-RNA binding regions of the core-associated VP6 were essential for BTV replication, while those associated with reVP6 were dispensable. Within the essential binding sites, residues that recognize BTV RNA preferentially, which are possibly necessary for genome recruitment and packaging, were identified. This study highlights the essential role of the orbivirus protein VP6 in genome packaging and replication.

## RESULTS

### Genomic ssRNA is not packaged in the viral capsid in the absence of VP6.

**FIG 1:**
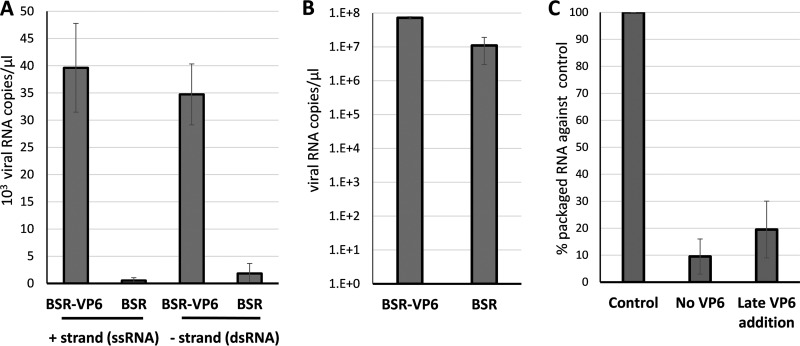
RNA packaging in viral particles relies on VP6. (A) A virus with truncated VP6 was used to infect BSR or BSR-VP6 cells. Twelve hours later, the viral particles were harvested and purified, and the positive (+) and negative (–) strands, representing genomic ssRNA and dsRNA, respectively, were measured by qRT-PCR. (B) BTV ssRNAs in the cell lysate from the experiment for which results are shown in panel A were measured by qRT-PCR. (C) A CFA assay was performed either by the original method (control), in the absence of VP6, or with VP6 added at a later time point. The assembled complex was purified and packaged, and viral RNA was measured by qRT-PCR. Mean values ± standard deviations are shown (*n* = 3).

During BTV replication, newly synthesized ssRNA segments are first packaged into the assembling viral cores, which, in turn, serve as templates for the synthesis of genomic dsRNA segments (5). VP6 has been shown to be an essential component of the primary replication complex and has been hypothesized to function in recruiting and packaging BTV RNA (9, 10, 13). Rescued BTV carrying a truncated VP6 protein was possible only in a VP6-complemented cell line, and although these rescued VP6-defective viruses could express BTV proteins upon infection, they assembled only a low level of particles, which lacked the viral genome, as visualized by electron microscopy (11). To confirm the correlation between a functional VP6 and RNA packaging, an available BTV strain with a truncated VP6 protein (triple stop codons introduced at residues 87 to 89) (16) was grown in a VP6-complemented BSR-derived cell line (BSR-VP6), and after 3 days, the recovered virion particles were used to infect both parental BSR cells and BSR-VP6 cells. Although these particles lacked the S9 RNA segment, which encodes VP6, they still contained VP6 protein incorporated from the BSR-VP6 cells used to recover the virus and thus were capable of synthesizing first-round ssRNAs, though not capable of completion of replication or second-round transcription following infection of wild-type (WT) BSR cells. Newly synthesized viral particles were harvested after 12 h, the result of only one replication cycle. The packaged RNA was quantified by reverse transcription-quantitative PCR (qRT-PCR), assaying for both positive and negative strands of S6 RNA with an S6 RNA segment-specific probe. While both the positive and negative strands of RNA molecules were present in the particles recovered from BSR-VP6 cells, particles from normal cells had <3% of the amount observed in particles grown in BSR-VP6 cells (Fig. 1A). This low-level signal is most likely residual RNA from the inoculum. These data confirm that in the absence of functional VP6, newly synthesized viral RNA segments are not packaged, a finding consistent with a role for VP6 in the packaging of viral RNA. As a control, we measured the BTV ssRNA in the cell lysate from BSR-VP6 or BSR cells infected with VP6-truncated particles (Fig. 1B). BTV ssRNA transcripts were synthesized abundantly in both cell lines, slightly more in BSR-VP6 cells than in WT BSR cells.

To investigate at which stage VP6 can affect virus assembly, we used the established *in vitro* cell-free assembly (CFA) assay (5). In this assay, the 10 ssRNA segments of BTV are first incubated with replicase complex proteins VP1, VP4, and VP6, followed by incubation with the inner capsid proteins VP3 and VP7, to assemble a core particle. We modified this assay for our study by either excluding VP6 or including it after the addition of VP3, so that it would not be incorporated into the core particle. The amount of RNA encapsidated was then quantified using RT-PCR as before. In the absence of VP6, ssRNA packaging was reduced to <10% of that with the control (Fig. 1C). Further, the addition of VP6 after VP3, which forms the inner layer of the core, failed to rescue RNA packaging. These data further suggest that VP6 plays a role in the early stage of genomic ssRNA packaging prior to inner core assembly.

### Identification of the RNA binding sites of VP6 and their impact on virus replication.

**FIG 2:**
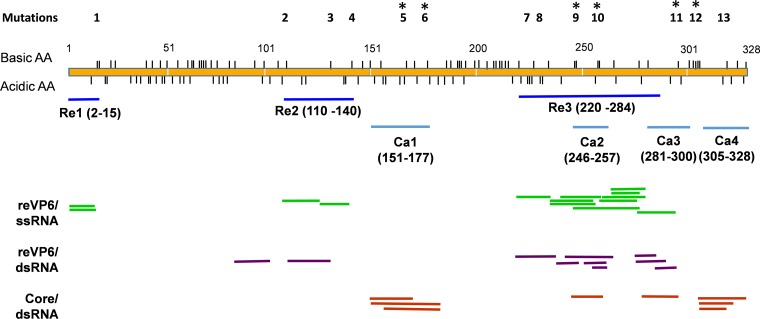
Regions of the BTV VP6 that contact RNA. The yellow bar represents the VP6 protein, with the vertical dashes denoting the positions of positively and negatively charged amino acids. The colored lines represent peptides identified in the RCAP analyses performed with recombinant VP6 protein (reVP6) and either single-stranded or double-stranded RNAs and with VP6 from purified cores. Details of the peptides are presented in Table 1. The three regions in reVP6 that contact RNAs are named Re1 to Re3. Regions in the core-associated VP6 that contact packaged genomic RNAs are named Ca1 to Ca4. Mutations were introduced on 13 specific charged sites (top), and the sites at which mutations had lethal effects are marked with asterisks.

VP6 has a high number of charged residues and readily binds nonspecific ssRNA and dsRNA *in vitro* (17, 18). We sought to identify the RNA binding regions in VP6 using a proteomics-based RCAP method. For the RCAP analysis, we used S10, the smallest of the BTV RNA segments, and a recombinant VP6 (reVP6) expressed in the baculovirus expression system. Two independent experiments, each with independent samples in triplicate, identified very similar RNA binding regions within reVP6 (Fig. 2), indicating that such an approach can reproducibly identify residues that contact RNA. None of the peptides were present in reaction mixtures lacking RNA, and the majority were also absent in control reaction mixtures that were not cross-linked with formaldehyde (Table 1). Three regions of VP6 were strongly associated with the binding of ssRNA: aa 2 to 15 (Re1), aa 110 to 141 (Re2), and aa 220 to 284 (Re3) (Fig. 2). To assess whether these *in vitro* RNA-binding sites are important for virus replication, six positively charged sites within VP6 (KR_14-5_, K_110_, K_131_, K_141_, KK_246-7_, RK_257-8_) were selected for substitution mutagenesis. To perturb the potential RNA binding affinity, each residue was replaced with glutamic acid (Glu; E) either individually (K_110_, R_131_, R_141_) or as a double substitution mutation (KR_14-5_, KK_246-7_, RK_257-8_). Each of these mutant segments, together with the other nine WT BTV RNA segments, was then transfected into BSR cells for virus recovery.

**TABLE 1:** Characteristics of the VP6 peptides assigned to contact RNA

Sample	*m*/*z*	Error (ppm)	Assign.Confid.^*a*^ (%)	Peptide sequence	Position (aa)	Modifications	Fold increase above area with no HCHO^*b*^
reVP6/ssRNA	655.39	4.7	97.8	SAAILLAPGDVIK	2–14	Acetylation, N-terminal	>10
reVP6/ssRNA	733.44	4.6	100	SAAILLAPGDVIKR	2–15	Acetylation, N-terminal	>10
reVP6/dsRNA	557.80	4.4	100	IHTAVGSGSGTK	83–94	None	>10
reVP6/ssRNA and reVP6/dsRNA	851.90	5.3	100	VGGGGGDADAGVGATGTNGGR	111–131	None	>10, 5.6
reVP6/ssRNA	608.34	0.5	100	WVVLTEEIAR	132–141	None	>10
Core/dsRNA	682.03	0.4	100	IDVYRDEVPAQIIEVER	151–167	None	>10
Core/dsRNA	789.68	2.4	100	IDVYRDEVPAQIIEVERSLQKELGISR	151–177	None	>10
Core/dsRNA	699.37	0.4	100	DEVPAQIIEVERSLQKELGISR	156–177	None	>10
reVP6/ssRNA and dsRNA	795.37	–3.6	100	EGVEEEKTSEEPAR	221–234	None	>10
reVP6/ssRNA and dsRNA	646.35	3.5	100	IGITIEGVMSQK	235–246	Oxidation of M9	>10
reVP6/ssRNA and dsRNA	545.80	1.6	100	GVMSQKKLLSMIGGVERKMA	241–260	Oxidation of M11	>10
reVP6/ssRNA, dsRNA, and core	609.85	1.7	100	KLLSMIGGVER	247–257	Oxidation of M5	>10
reVP6/ssRNA and dsRNA	545.80	2.2	100	LLSMIGGVER	248–257	Oxidation of M4	5.2, 5.2
reVP6/ssRNA and dsRNA	647.34	1.3	100	ESAVMLVSNSIK	266–277	Oxidation of M5	>10
reVP6/ssRNA and dsRNA	588.32	1.5	100	ESAVMLVSNSIKDVVR	266–281	Oxidation of M5	>10
reVP6/ssRNA and dsRNA	554.94	–0.1	100	ATAYFTAPTGDPHWK	282–296	None	>10
Core/dsRNA	706.69	–1.6	83.9	ATAYFTAPTGDPHWKEVAR	282–300	None	>10
Core/dsRNA	669.85	0.1	100	NILAYTSTGGDVK	307–319	None	>10
Core/dsRNA	784.89	–0.8	100	NILAYTSTGGDVKTE	307–321	None	>10
Core/dsRNA	853.12	1.1	100	NILAYTSTGGDVKTEFLHLIDHL	307–329	None	>10
reVP6/ssRNA	473.94	4.5	100	IGITIEGVMSQKK	235–247	None	>10
reVP6/ssRNA	609.85	–2.6	82.9	LLSMIGGVERK	248–258	None	>10
reVP6/ssRNA	558.82	2.9	93.5	VSNSIKDVVR	272–281	None	>10

a Confidence of peptide assignment from collision-induced peptide fragmentation.

b The area of the peptide peak relative to the area for the reaction performed without formaldehyde.

**FIG 3:**
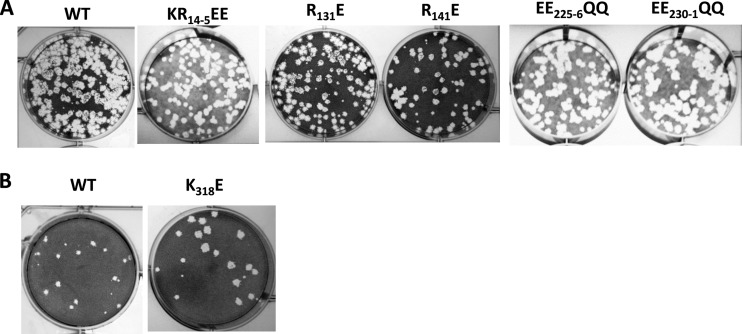
Plaque formations of WT and mutant viruses.

Mutations in Re1 (KR_14-5_EE) or Re2 (K_110_E, R_131_E, and R_141_E) did not affect virus recovery; recovered mutant viruses produced plaques similar in size to those of the parental virus (Fig. 3A). DNA sequencing confirmed the presence of the mutations within S9 for all recovered viruses and the absence of compensatory mutations elsewhere. In contrast, the double mutations located within the RNA binding region Re3 (KK_246-7_EE and RK_257-8_EE) prevented virus recovery despite several independent attempts. To examine this further, two pairs of negatively charged residues from a conserved Glu-rich motif (EE-K-XX-EE) in the same Re3 region were also mutated to neutrally charged glutamine (EE_225-6_QQ and EE_230-1_QQ). Both mutant constructs were then tested for their impact on virus recovery by RG. In both cases, the virus was successfully recovered, suggesting that positively charged residues in region Re3, but not negatively charged residues, are important for viral infection. The positively charged residues located in RNA binding region Re3 are thus essential for virus replication.

Although VP6 binds both ssRNA and dsRNA (17), it is not known if the sites concerned are the same. To investigate this, BTV genomic dsRNA segments were isolated from BTV-infected cells, and the RCAP analysis with reVP6 was repeated using dsRNA. The dsRNA-binding regions identified were similar to those that bind ssRNA (Fig. 2, Table 1), suggesting that, in the absence of other viral proteins, VP6 binds ssRNA and dsRNA in a similar manner.

### Identification of VP6-RNA binding regions within the viral capsid and their importance for virus replication.

Recombinant VP6 has multiple regions that contact ssRNA and dsRNA. However, VP6 found in the virus cores is in contact with capsid protein, and this could impact RNA binding. To examine this, we purified mature cores and subjected them to RCAP analysis (Fig. 2). Peptides from several core-associated proteins were identified, and those from VP6 were assigned based on collision-induced fragmentation of each peptide. Within VP6, four regions were found to contact the encapsidated genomic dsRNA: aa 151 to 177 (Ca1), aa 246 to 257 (Ca2), aa 281 to 300 (Ca3), and aa 305 to 328 (Ca4). Notably, only Ca2 overlaps with RNA-binding region 3, which was found when the analysis was done with reVP6. Thus, core-associated VP6 has a binding profile different from that of recombinant VP6. To map the essential binding sites, a number of positively charged residues in the identified Ca regions were targeted for site-directed mutagenesis and virus recovery using the RG system. Five site-specific mutations were introduced: R_167_E and R_177_E, located in region Ca1, K_296_E in region Ca3, and KRR_303-5_EEE and K_318_E in region Ca4. Among the five mutations introduced into VP6, only K_318_E permitted the recovery of viruses with normal plaque sizes (Fig. 3B). All the other mutations abrogated virus recovery. In agreement with previous data, the two positively charged sites (KK_246-7_EE and RK_257-8_EE) in the Ca2–Re3 shared region (Ca2/Re3) were lethal, confirming these regions as critical for virus replication. Residues RK_208-209_ of VP6, which were previously proposed to contact BTV RNA by bioinformatics analysis, were not identified by RCAP (14). In contrast to the Ca mutants, an RK_208-209_EE VP6 mutant did not perturb virus recovery, demonstrating that RCAP is a more precise method for recognizing RNA-contacting residues.

Previous data have suggested that certain RNA sequences in the BTV genome, especially in the smaller RNA segments, act as packaging signals that mediate RNA assembly through RNA-RNA interaction (1, 15). To ensure that the mutations introduced into the S9 RNA sequence encoding VP6 did not affect RNA packaging, the lethal mutations described above were also rescued using an RG system in VP6-complemented BSR-VP6 cells (9). Viruses with mutations in all six Ca regions produced the virus in BSR-VP6 cells. Furthermore, the rescued virus failed to grow when passaged on normal BSR cells. These results confirmed that the lethal effect of these mutations was due to changes in VP6 protein, not to a change in the packaging signals in S9 RNA.

**FIG 4:**
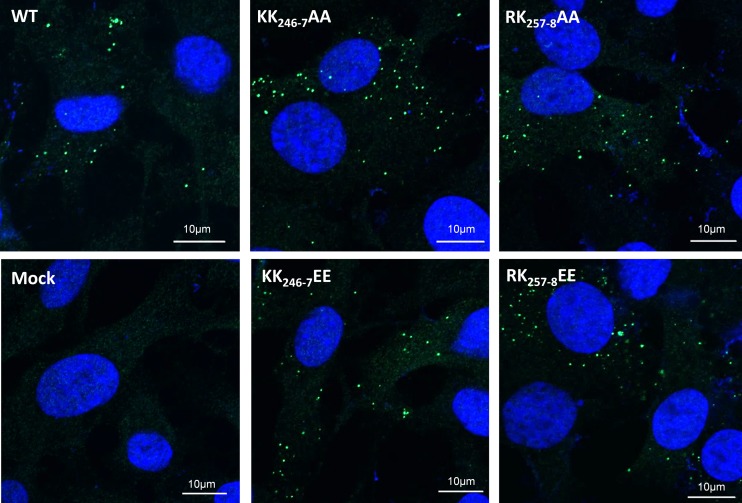
Mutant VP6 is expressed similarly to wild-type (WT) VP6. Four mutants (KK_246-7_AA, KK_246-7_EE, RK_257-8_AA, and RK_257-8_EE) and WT VP6 encoding S9 RNAs were used to transfect BSR cells. Protein expression and localization were monitored by immunofluorescence staining and confocal microscopy. Green, VP6; blue, Hoechst staining.

To exclude the concern that substitutions of negatively charged residues may influence the protein conformation or isoelectric point, five lethal mutations were also redesigned to change the positively charged R or K into alanine (Ala; A) (R_167_A, R_177_A, KK_246_-_7_AA, RK_257_-_8_AA, and K_296_A). None of these mutants was recovered following RG transfection in the normal BSR cells (data not shown). However, when S9 RNA carrying critical Ca2/Re3 region mutations (KK_246_-_7_EE, RK_257_-_8_EE, KK_246_-_7_AA, and RK_257_-_8_AA) was transfected into BSR cells, all the mutant VP6 proteins were expressed in the cells, and the localization was not different from that of WT VP6 (Fig. 4).

**FIG 5:**
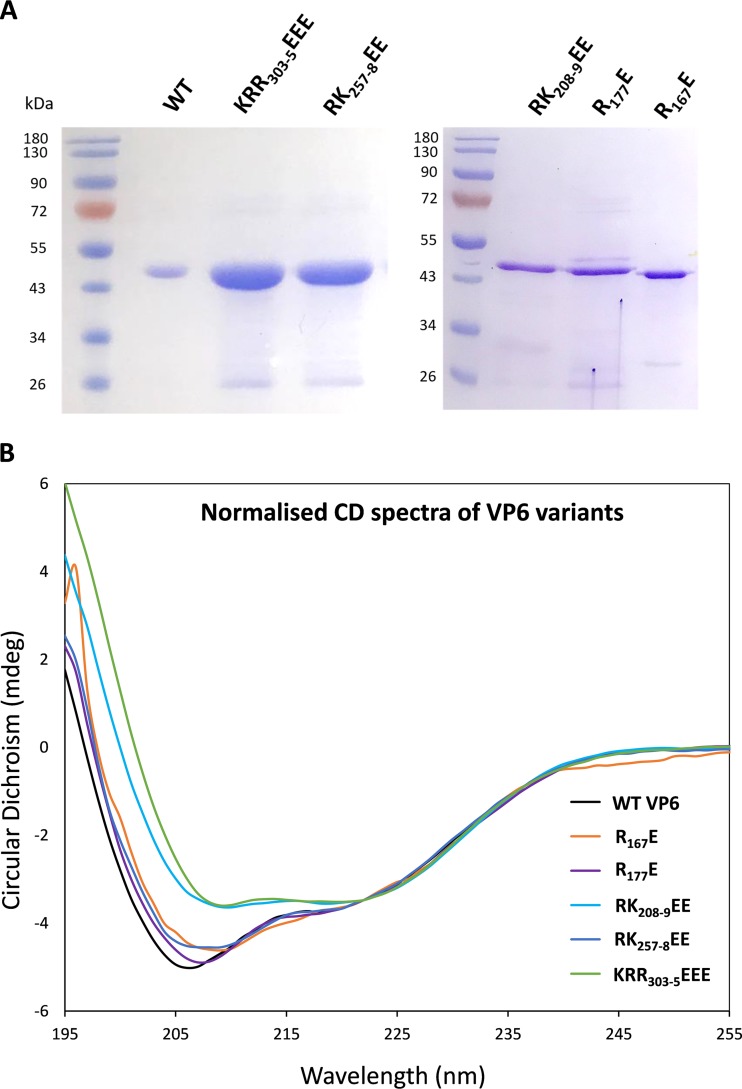
Conformational analysis of mutant reVP6. (A) Five mutant reVP6 proteins were expressed in E. coli and analyzed by SDS-PAGE together with wild-type (WT) VP6, followed by Coomassie blue staining. The sizes on the protein ladder are indicated. (B) CD spectra of VP6 mutants normalized to those of wild-type VP6. Each mutant and wild-type VP6 is indicated by a different color.

To ensure that no gross conformational change in the mutation mapped as critical, several mutant VP6 proteins were expressed in E. coli, purified, and analyzed by gel electrophoresis and for secondary structure. Each mutant VP6 exhibited the same mobility as WT VP6 (Fig. 5A), and when analyzed by circular dichroism (CD) spectroscopy, all showed similar spectra except for slight differences from the WT for RK_208-9_EE and KRR_303_-_5_EEE. However, the relative values of the α-helix and beta sheet did not vary beyond the variance of the assay (Fig. 5B and Table 2). We calculated the secondary-structure elements using tool K2D3. The change in the percentage of individual secondary-structure components was clearly shown not to be significant.

**TABLE 2:** Conformational analysis of mutant reVP6

VP6	Helix/loop (%) (α, π, 3,10 helix, loop)	Beta strands (%)	Turn (%)	RMSD^*a*^
WT	48.0	36.6	15.5	0.0467
R_167_E	47.7	37.0	15.3	0.0499
R_177_E	47.3	37.1	15.5	0.0506
RK_208_-_9_EE	47.4	37.2	15.5	0.0493
RK_257_-_8_EE	47.8	36.8	15.4	0.0478
KRR_303_-_5_EEE	47.3	37.3	15.4	0.0486

a RMSD, root mean square deviation.

Taken together, these data suggest that mutations introduced into VP6 to probe its RNA binding function, studied here, did not induce major conformational changes in any of the mutants described.

### The lethal VP6 mutant lacks preferential binding affinity for viral RNA.

**FIG 6:**
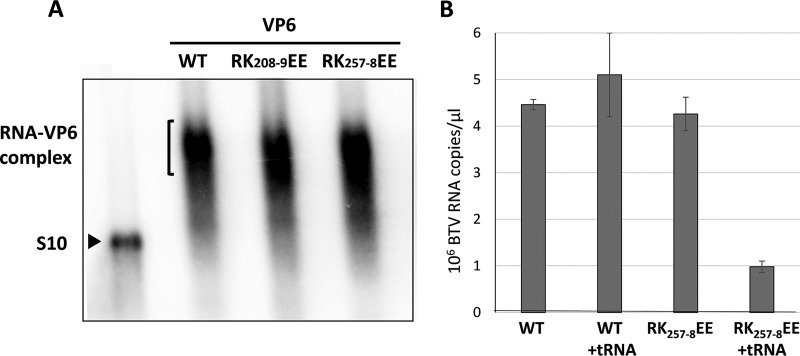
VP6 shows a BTV RNA binding preference. (A) RK_208-9_EE mutant, RK_257-8_EE mutant, or WT VP6 was incubated with ^32^P-labeled BTV S10 ssRNA for interaction. The shifting of the VP6-RNA complex was observed by EMSA using a 0.8% agarose gel and TBE buffer before analysis with s phosphorimager. (B) WT or RK_257-8_EE reVP6 was bound to Ni beads and was incubated with BTV ssRNA S10 in the absence or presence of a 100-fold quantity of tRNA (+tRNA). The bound RNA was then eluted and quantified by qRT-PCR. Mean values ± standard deviations are shown (*n* = 3).

BTV S10 ssRNA was previously found to be critical for efficient RNA packaging (1, 15, 19) and was therefore used as the source of RNA to investigate the preference of VP6 for RNA binding. VP6 binding to S10 RNA was examined using ^32^P-labeled S10 RNA by a gel electrophoretic mobility shift assay (EMSA). Three recombinant VP6 proteins were tested: a replication-competent mutant, RK_208-9_EE; a replication-incompetent mutant, RK_257-8_EE, which is located within the Ca2/Re3 region; and WT reVP6. In the EMSA, all three proteins exhibited strong band retardation, plausibly a measure of the nonspecific RNA binding function described above (Fig. 6A). To investigate preferential binding by VP6, an alternate, competition method of RNA-protein interaction was used. S10 ssRNA was exposed to Ni-nitrilotriacetic acid (NTA) agarose beads coated with His-tagged WT reVP6 or RK_257-8_EE mutant in the presence or absence of an excess of yeast tRNA, and after pulldown, the bound RNA was quantified by qRT-PCR. The amount of RNA bound by WT reVP6 was not significantly affected in the presence of excess yeast tRNA, while for the RK_257-8_EE mutant, the presence of excess tRNA significantly reduced the level of S10 RNA bound (Fig. 6B).

**FIG 7:**
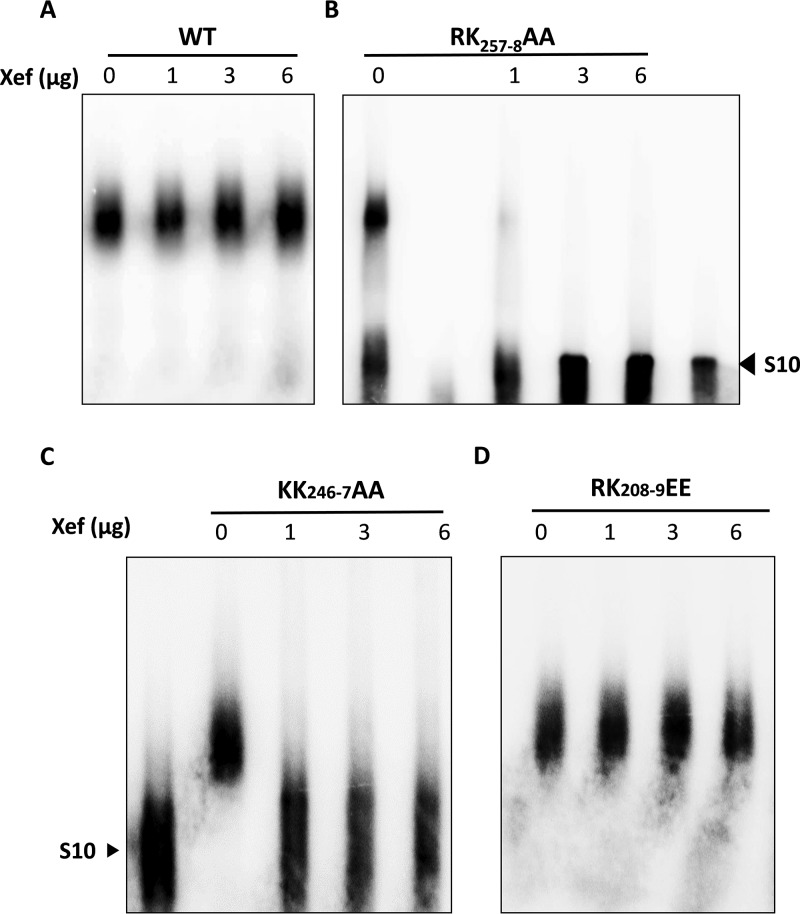
Mutations in the Ca2/Re3 region destroy BTV RNA preferential binding. A competition assay was performed using 1 μg of WT (A), RK_257-8_AA (B), KK_246-7_AA (C), or RK_208-9_EE (D) reVP6 and 0.1 μg of ^32^P-labeled BTV S10. *Xenopus* elongation factor (Xef) mRNA was added in the quantities indicated. The size of free S10 RNA is indicated.

To confirm this in a quantitative manner, we performed a competition assay in the presence of different quantities of nonspecific RNA (Fig. 7). To exclude the possibility that the difference in binding was due to the small size of the competitor tRNA, we used a longer ssRNA encoding *Xenopus* elongation factor 1α as a nonspecific RNA. Further, to ensure that the RNA binding was not influenced by changes in charge, we expressed the mutant RK_257-8_AA in E. coli, purified it, and utilized it for EMSA. The data showed that RK_257-8_AA reVP6 had much lower preferential binding with BTV RNA than WT reVP6 (Fig. 7A and B). Moreover, when an additional positively charged site, RK_246-7_, within the same Ca2/Re3 region was mutated to Ala, it also exhibited a significant effect on the preference of VP6 for BTV RNA (Fig. 7C). However, the replication-competent RK_208-9_EE mutant exhibited gel shifting patterns similar to those of WT reVP6 (Fig. 7D). These results indicate that the Re3/Ca2 region in VP6 is part of a preferential BTV RNA binding site.

### Mutant VP6 is unable to package viral RNAs.

**FIG 8:**
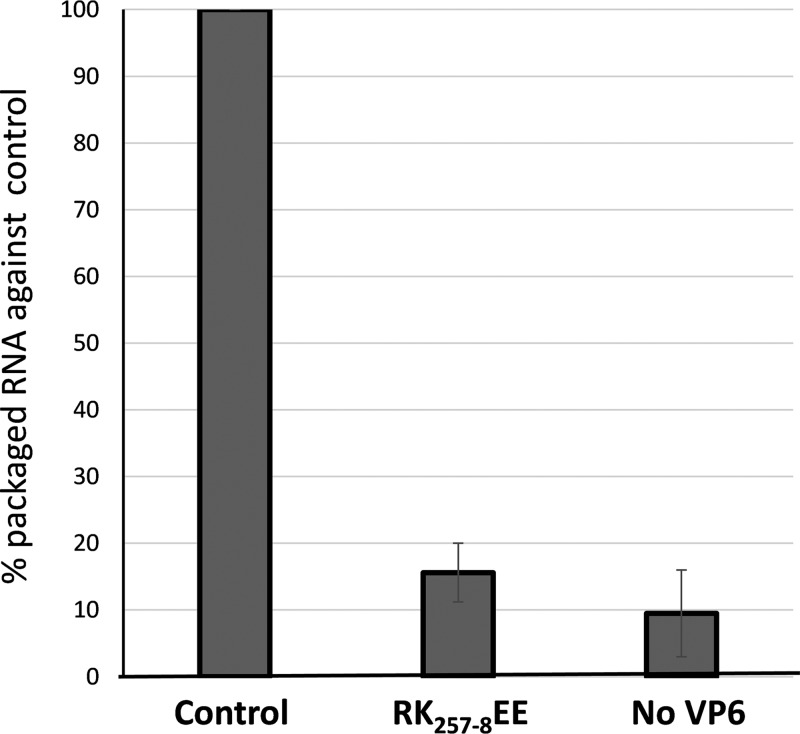
Mutation on VP6 prohibits genomic RNA packaging. A CFA assay was performed in the presence of WT VP6 (control) or RK_257-8_EE VP6 or in the absence of VP6. The assembled complex was purified and the packaged viral RNA measured by qRT-PCR. Mean values ± standard deviations are shown (*n* = 3).

**FIG 9:**
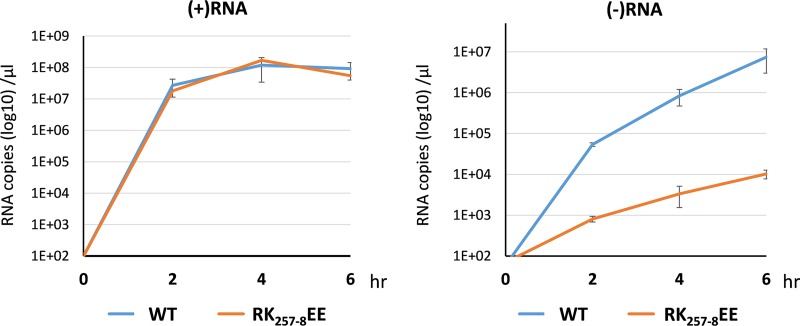
A VP6 mutant virus is deficient at producing the dsRNA genome. The VP6 RK_257-8_EE mutant virus was used to infect BSR cells (RK_257_-_8_EE) (red line) or BSR-VP6 cells (WT) (blue line). Total cytoplasmic RNA was harvested at 0, 2, 4, and 6 hpi. The positive-strand (+) and negative-strand (–) RNA, representing genomic ssRNA (left) and dsRNA (right), respectively, were measured by qRT-PCR. Mean values ± standard deviations are shown (*n* = 3).

The Ca2/Re3 region binds both ssRNA *in vitro* and dsRNA in the viral capsid. Further, it exhibits a preference for BTV RNA, consistent with a key role in the recruitment of ssRNA into assembling capsids. To confirm this, we employed the *in vitro* CFA assay, which had already demonstrated that RNA packaging was dependent on VP6. Making use of the RK_257-8_EE VP6 mutant, WT VP6 (positive control), or no VP6 (negative control), the level of incorporated genomic RNAs in the *in vitro*-assembled cores was measured by qRT-PCR. RNA packaging was significantly reduced from that with the WT when the RK_257-8_ mutant protein was present, almost to the background level of incorporation observed in the absence of VP6 (Fig. 8). To assess if a similar effect occurs in infected cells, the RK_257-8_EE mutant virus grown in the BSR-VP6 helper cell line was used to infect the parental BSR cell line for 6 h, and total RNA was extracted from 0 to 6 h postinfection (hpi). As a control, BSR-VP6 cells, which are permissive for the growth of the mutant, were also infected with the same virus preparation, and RNA was extracted. All RNA samples were analyzed by qRT-PCR using primers specific for the detection of positive- or negative-sense RNA, as described above. In this analysis, quantification of the negative strand acts as a marker for dsRNA synthesis, as described previously (5, 11). During the infection period, similar quantities of positive-sense BTV RNA were produced by the mutant virus in both cell lines. However, in the parental BSR cells, the level of negative-sense RNA synthesis was ∼1,000-fold lower than that with the parental virus at 6 hpi (Fig. 9). These data are consistent with VP6 acting in the packaging of BTV RNA into the core via preferential recognition by the Ca2/Re3 region.

## DISCUSSION

Previous reports have implicated VP6, a protein with clusters of charged residues, in the packaging of the viral genomic RNA, but precise mapping of the binding sites and their contribution to virus replication was not reported (8, 11, 13, 14, 17). We used a robust peptide-mapping method, RCAP, which could identify interactions between VP6 and the viral genome in the natural state, leading to the identification of four (Ca) VP6-BTV RNA binding sites. Compared to binding sites mapped by the binding of recombinant VP6 (reVP6) to RNA *in vitro*, several regions were distinct, suggesting that the binding of RNA by VP6 contains both specific and nonspecific elements. Mutations in the Ca RNA binding sites targeting positively charged residues (R_167_, R_177_, KK_246-7_, RK_257-8_, K_296_, and KRR_303-5_) prevented virus recovery irrespective of the charge status of the mutated residues. These six sites are highly conserved among the VP6 proteins of different BTV serotypes and the related orbiviruses epizootic hemorrhagic disease virus (EHDV) and African horse sickness virus (AHSV). This suggests that the interaction between VP6 and RNA within the core is invariant, in agreement with a key functional role.

Recently, a specific site of VP6 (aa 276 to 287) was reported to interact with the inner layer capsid protein VP3, an interaction that was essential for virus assembly (8). This interaction site is distinct from the RNA interaction sites reported here, suggesting that it may act to bridge the captured genomic RNA with the assembling capsid. Some viruses, such as bacteriophage MS2, use their coat protein to package genomic RNA through specific signals (20, 21), but for complex, multilayered capsid viruses, such as orbiviruses, genome packaging is likely to engage more than one viral protein. The two different kinds of interactions that are seen in BTV, VP6-VP3 and VP6-RNA, are consistent with this hypothesis.

In contrast, RCAP analysis of the *in vitro* RNA-binding regions of reVP6 were largely not specific, a conclusion supported by the EMSA data. The amino-terminal region (Re1) of reVP6 is also conserved among the orbiviruses, but changing the only positively charged residues in this region, KR_14-5_, did not influence virus replication, suggesting that this conservation of amino acid sequence is due to other functional reasons. The disorder prediction of VP6 structure showed that the N-terminal part of VP6 was more disordered than the C-terminal part, suggesting that the N terminus is highly mobile, with nonspecific binding, while the C terminus has a conserved function, which was consistent with our findings (22). Previous studies suggested that only the hexameric form of VP6 could perform helicase activity *in vitro*, although monomeric VP6 could still bind RNA (14, 17). However, it is still not known what form of VP6 exists within the viral capsid and in cells when it functions as an RNA binding protein. The relationship between VP6 oligomerization and its different functions requires further investigation.

One region revealed by the comparative RCAP analysis (aa 246 to 257; amino acid sequence KKLLSMIGGVERK) was associated with RNA binding both *in vitro* and in the viral capsid. This region has two sites, each with two positively charged residues, KK_246-7_ and RK_257-8_, and both were shown to be essential for viral replication. The same mutations led to VP6 losing its preference for BTV RNA in competition binding assays, suggesting that the region is part of a discriminatory mechanism that selects BTV RNA over the cellular pool. This observation of preferential binding could be due to the region of VP6 recognizing certain sequences or secondary structures in the viral RNA segments. However, the precise sequence(s) that VP6 binds during packaging and the question of whether VP6 plays an active role in RNA complex formation remain to be investigated.

In an earlier report, a cell-free assembly assay was established whereby the sequential mixing of replicase proteins VP1, VP4, and VP6, together with BTV ssRNAs, followed by the sequential addition of the inner capsid proteins VP3 and VP7, resulted in the successful packaging of ssRNAs into a core structure (5). In the absence of VP6 during sequential assembly, packaging of ssRNAs is not observed, again in agreement with a role for VP6 in the packaging of ssRNA into the BTV core structure. When a single-cycle infection assay was performed with the RK_257-8_ mutant virus, amplified in a helper cell line, although it was still able to synthesize positive-sense viral ssRNAs, only low levels of negative-sense RNAs were detected, insufficient to support viral rescue. These data may indicate marginal VP6 activity, below the threshold required for growth, or the residual activity of the VP6 incorporated from the BSR-VP6 cells used for mutant virus recovery. These data clearly indicate that VP6 is critical in an early function, such as selective binding to BTV RNA, and that the region encompassing aa 246 to 257 is a required motif.

Viral protein helps RNA virus to correctly its genome package through diverse mechanisms. Besides the bacteriophage coat protein mentioned above, dsRNA bacteriophages of the *Cystoviridae* family contain a hexameric ATPase, P4, that serves as an RNA-packaging motor (23, 24). Recently, an alphatetravirus was found to have a small encapsidated protein, P17, that specifically binds to viral RNA and assists in RNA packaging (25). Orbivirus VP6, despite some similarities with other packaging proteins, appears to have its unique mechanism.

Previous studies have suggested an ATPase motif and a helicase motif of VP6 based on *in vitro* functional analysis (14). K_110_, the previously reported ATPase motif, was in the RCAP-identified RNA binding region (Re2). However, changing this site did not influence virus replication. E_157_, a site in the previously suggested helicase (DEAD box), also failed to show any influence on virus growth (unpublished observation). Both these sites are conserved within BTV but are not shared with EHDV or AHSV. Studies have shown that the ATP-driven duplex unwinding function is not necessarily the primary mechanism of helicase proteins (26, 27). Helicase protein can serve as a translocator without unwinding the duplex (28–31). Further investigation is required to clarify the roles of the ATPase and helicase activities of VP6 during genome packaging.

It is highly likely that RNA segments interact with each other in a sequential manner prior to the packaging of the RNA complex into the capsid (1, 5, 15). In a recent report, we showed that RNA segment assortment is based mainly on RNA-RNA interactions via specific sequences (32). It is possible that VP6 is also actively involved in assortment, but our current data do not support this notion.

Structural analysis showed that VP1 and VP4 could be encapsidated within the core-like particles of VP3 and VP7, while VP6 alone could not be encapsidated without viral RNA (7). It can be hypothesized that not only is VP6 essential for genome packaging, but also, perhaps reciprocally, that genomic RNA plays a role in VP6 encapsidation. More-detailed structural and functional studies are required to elucidate further the protein-RNA arrangements in the viral capsid and precisely how these interactions promote genome packaging during capsid assembly.

## MATERIALS AND METHODS

### Virus, plasmids, mutagenesis, and RNA transcript synthesis.

BTV-10 VP6 was used for mutational analysis and reverse genetics (14, 17). BTV-1 (GenBank accession numbers FJ969719 to FJ969728), the VP6 of which is fully exchangeable with that of BTV-10, was used in RCAP for viral capsids. All mutations of VP6 were generated by site-directed mutagenesis, and sequences of these mutations and primers are available upon request. Transcripts for reverse genetic analyses were prepared using the mMACHINE T7 transcription kit (Thermo); RNA transcripts for gel shifting and competition assays were prepared using T7 polymerase (Thermo), following the manufacturers’ instructions.

### reVP6 expression and purification.

The expression and purification of recombinant VP6 (reVP6) of BTV-10 by use of baculovirus in the Sf9 cell line have been described previously (14). Additionally, His-tagged wild-type (WT) and mutant reVP6 proteins were expressed in E. coli strain BL21(DE3) pLysS. The His-tagged reVP6 proteins were purified using Ni-NTA affinity purification, eluting the purified protein with a buffer comprising 20 mM Tris-HCl, 200 mM NaCl (pH 7.4), and 250 mM imidazole. Imidazole was then removed by buffer exchange with Sephadex G-75 columns (GE Healthcare).

### RCAP.

The RCAP assay was carried out as described previously (33, 34). For analysis of the binding of recombinant VP6 to RNA, 1 mol of RNA was added to 2 mol of recombinant protein. The molar ratio of RNA was kept low to decrease nonspecific protein cross-linking to RNA. Formaldehyde was then added to a final concentration of 0.1%, and the mixture was incubated for 10 min at room temperature. Glycine was added to a concentration of 0.2 M for 10 min to quench additional cross-linking. The cross-linked protein-RNA complexes were digested using sequencing-grade trypsin (Trypsin Gold; Promega) for 16 h at a 1:20 (wt/wt) ratio of trypsin to capsid. RNA-peptide complexes were then selectively precipitated using a final concentration of 3 M lithium chloride and centrifugation at 16,000 × *g*. The peptide-RNA conjugates were reversed by a 1-h incubation at 70°C. Parallel control reactions to assess background signals were performed without the addition of formaldehyde or, in the case of the recombinant protein, without RNA. RCAP peptides were analyzed using an Orbitrap Elite hybrid ion trap mass spectrometer equipped with an electrospray ionization source (Thermo Fisher Scientific). The peptides were resolved using a Dionex UltiMate 3000 high-performance liquid chromatograph (HPLC) with a 1- by 150-mm Zorbax 300SB C_18_ column (Agilent) and were eluted using a linear gradient of 2% to 45% acetonitrile in water with 0.1% formic acid over 90 min with a flow rate of 50 μl/min. Tandem mass spectra were obtained using collision-induced dissociation in a data-dependent manner. Raw mass spectral data files were converted to Mascot generic format and were analyzed using SearchGUI (35). Spectra were searched against a database of BTV proteins concatenated with the cRAP database (36). Unspecific enzyme cleavage and a mass tolerance of 10 ppm were used. Search results were compiled and were visualized using PeptideShaker (37), and results were exported as a CSV file for automated processing with custom KNIME workflows (38).

### Immunofluorescence staining and confocal microscopy.

BSR cells were transfected with 800 ng of VP6-capped S9 RNA containing wild-type and mutant VP6 together with 800 ng of NS2 encoding capped S8 RNA by using Endofectin (GeneCopoeia), according to the manufacturer’s instructions. At 24 h posttransfection, cells were fixed with a 4% paraformaldehyde (Sigma) solution, permeabilized with 0.5% Triton X-100 (Sigma), blocked with 1% bovine serum albumin (BSA; Sigma), and subsequently stained using homemade rabbit anti-NS2 and guinea pig anti-VP6 as primary antibodies and Alexa Fluor 546-conjugated anti-rabbit and Alexa Fluor 488-conjugated anti-guinea pig secondary antibodies (Thermo Fisher Scientific). Nuclei were stained using Hoechst 33342 (Thermo Fisher Scientific). Images were acquired using a 100× oil objective and a Zeiss Axiovert LSM510 confocal microscope supplied with LSM510 software.

### CD spectra of reVP6.

The circular dichroism (CD) spectra of reVP6 were recorded in CD buffer (20 mM Na_2_HPO_4_, 100 mM NaCl [pH 7.4]) at 20°C. The far-UV CD spectral data were collected from 260 to 195 nm with a 0.5-mm rectangular cell path length at 20°C on the Applied Photophysics Chirascan and Chirascan Plus spectrometers (Leatherhead, UK) attached to a Peltier unit (TC125; Quantum Northwest). The UV and CD spectra were smoothed (window factor of 4, Savitzky-Golay method) and analyzed using Origin, v6, and APL Pro-Data Viewer, v4.2.15. The percentage of secondary structural units (α-helix and beta strand) of protein from the experimental CD spectra was calculated using the K2D3 program as described previously (39).

### EMSA using ^32^P-labeled RNA.

RNA labeling and the electrophoretic mobility shift assay (EMSA) were performed as described previously (40). A typical binding assay mixture of 20 μl contains 3 μg of reVP6 in the RNA binding buffer (2 mM MgCl_2_, 60 mM KCl, 100 mM NaCl, 20 mM HEPES [pH 7.5], 10% glycerol, 1 U of RNasin [Promega]). Radiolabeled BTV S10 was heated to 65°C for 2 min, and the mixture of VP6 in binding buffer was added and incubated for 30 min at 4°C. Samples were then analyzed on 0.8% agarose gels in TBE (Tris-borate-EDTA, comprising 89 mM Tris [pH 7.4], 89 mM boric acid, 2.5 mM EDTA). The gels were dried and analyzed by autoradiography or by use of an Amersham Biosciences PhosphorImager. For the competition assay, 18 nM radiolabeled S10 RNA was incubated with 4 μM reVP6 in the presence of 1, 3, or 6 μg (85, 255, or 510 nM) of nonspecific *Xenopus* elongation factor mRNA (Thermo Scientific). The mixture was analyzed as described above.

### Reverse genetics.

Mutations in the cDNA of S9 RNA that encodes VP6 were generated using site-directed mutagenesis (sequences available upon request), together with the other 9 BTV genome segments that were used to transfect BSR cells or a BSR cell line that stably expresses VP6 (BSR-VP6), as described by Boyce et al. (41). Cytopathic effect (CPE) was monitored after 3 days, and the mutations in S9 in the recovered viruses were confirmed by RT-PCR and sequencing.

### Plaque assay.

WT and mutant viruses were diluted, applied to BSR cell monolayers at a multiplicity of infection (MOI) of 0.01 to 0.1, and covered by an Avicel overlay as described by Matrosovich et al. (42). Cells were fixed with formaldehyde and the plaque size monitored after 3 days.

### Single-cycle replication assay.

WT BTV, the virus with truncated VP6, and VP6 mutant viruses were used to infect BSR cells or BSR cells that stably express VP6 (BSR-VP6). The cells were harvested at 12 h postinfection (hpi), and viral particles were purified as described previously by Matsuo and Roy (11). The purified virus was treated with RNase A to remove nonpackaged RNA. The RNA was extracted using a viral RNA purification kit (Thermo Fisher Scientific). The positive- and negative-sense RNA, representing genomic ssRNA and dsRNA, respectively, were measured by qRT-PCR using viral RNA-specific primers (sequences available upon request) (43). To measure dsRNA synthesis, cell lysates were collected at different time points and were immediately frozen at −80°C, and the quantities of ssRNA and dsRNA in the cell lysate were similarly measured by qRT-PCR.

### *In vitro* CFA assay.

The BTV *in vitro* cell-free assembly (CFA) assay has been described previously (5). Briefly, the 10 segments of ssRNA were incubated with VP1, VP4, and VP6 for 30 min, followed by the addition of VP3 and VP7 sequentially with a 1.5-h incubation after each addition. As a control, the VP6 protein was either left out of the assembly reaction mixture or added after VP3. To avoid the VP6 protein being translated from the S9 of the 10 ssRNAs, we used a vaccine strain S9 RNA, which does not translate VP6. The samples were then subjected to a 15% to 65% sucrose gradient, and the packaged RNA was quantified by qRT-PCR.

### RNA binding assay.

Five micrograms of purified WT or mutant His-tagged VP6 was bound with 30 μl of Ni-NTA agarose beads (Thermo) in a buffer of 150 mM NaCl and 40 mM Tris-HCl (pH 8.0). The protein-coated beads were then incubated with 0.3 μg (1.1 pmol) of BTV S10 RNA in the presence or absence of 30 μg (1 nmol) of yeast tRNA. After extensive washes, the bound RNA was eluted by heating and quantified by qRT-PCR.

### qRT-PCR.

The extracted viral RNA from the single-cycle replication and CFA assays was subjected to RT using specific BTV S6 forward or reverse primers, followed by qPCR, using SYBR green master mix (LabTech) and S6 primers (43). RNA eluted from the VP6-RNA bead-binding assay was quantified using specific BTV S10 primers.
